# “You’re in an Image of a Man but Not a Man”: A Qualitative Analysis of Intersectional Stigma Among Men with HIV Experiencing Subfertility in Rural Southwestern Uganda

**DOI:** 10.1007/s10461-025-04611-3

**Published:** 2025-01-17

**Authors:** Madeline C. Pratt, Moran M. Owembabazi, Alex T. Menninger, Eunice Kanini, B. Rosemary Kansiime, Patricia M. Smith, Janet M. Turan, Lynn T. Matthews, Esther C. Atukunda

**Affiliations:** 1 Department of Medicine, Division of Infectious Diseases, University of Alabama at Birmingham Heersink School of Medicine, Birmingham, Alabama USA; 2 Mbarara University of Science and Technology, Global Health Collaborative, Mbarara, Uganda; 3 University of North Carolina Chapel Hill, Gillings School of Global Public Health, Chapel Hill, NC USA; 4 Department of Health Policy and Organization, School of Public Health, University of Alabama at Birmingham, Birmingham, Alabama USA

**Keywords:** HIV, Safer conception, Fertility, Uganda, Stigma, Qualitative methods

## Abstract

Many men with HIV (MWH) want to have children and may encounter HIV- and infertility-related stigma experiences. Integration of reproductive health and HIV care for men is rare. When available, safer conception care focuses on HIV prevention but lacks fertility support. We conducted qualitative in-depth interviews in Uganda with 30 MWH who desired more children and self-reported no partner pregnancy after 12 or more months of conception attempts. We separately interviewed 10 female partners. Interviews explored stigma experiences and factors impacting engagement in HIV and reproductive care. We used vignettes to elicit responses to stories of couples experiencing challenges of HIV and subfertility. The study team discussed, coded, and analyzed data from individual participant interview transcripts, inductively identifying emergent themes. The following overarching themes emerged: (1) Reproductive goals often take priority over HIV prevention among HIV-affected couples in this context, influenced by multi-level subfertility stigma in society. (2) MWH may pursue behaviors that increase risk of HIV transmission to meet their reproductive goals. (3) Men and women are eager to maintain their primary partnerships, prevent HIV transmission, and meet their reproductive goals with guidance from healthcare providers. Further research is needed on the causes of subfertility and infertility among HIV-affected couples in East Africa to better support their conception goals. Additionally, studies on the intersection of HIV and infertility stigma in high-fertility, high-HIV prevalence areas are essential for designing interventions that meet couples’ social, emotional, and medical needs.

## Resumen

Muchos hombres con VIH desean tener hijos, pero enfrentan el estigma relacionado con el VIH y la infertilidad. La integración del VIH y la atención reproductiva para los hombres es poco común. Realizamos entrevistas en profundidad con 30 hombres con VIH en Uganda que deseaban tener más hijos, pero no habían logrado el embarazo después de 12 meses o más de intentarlo, y entrevistamos por separado a 10 parejas femeninas. Las entrevistas exploraron el estigma y los factores que afectan la participación en el VIH y la atención reproductiva. Surgieron tres temas principales: (1) Los objetivos reproductivos a menudo tienen prioridad sobre la prevención del VIH, influenciados por el estigma social en torno a la subfertilidad. (2) Los hombres con VIH pueden participar en conductas que aumentan el riesgo de transmisión del VIH para alcanzar los objetivos reproductivos. (3) Tanto los hombres como las mujeres desean mantener relaciones, prevenir la transmisión del VIH y alcanzar los objetivos reproductivos con apoyo de atención médica. Se necesita más investigación sobre las causas de la subfertilidad y la intersección del estigma del VIH y la infertilidad en áreas con alto índice de VIH para mejorar las intervenciones para las necesidades sociales, emocionales y médicas de las parejas.

## Introduction

Engaging and retaining men in HIV care reduces HIV infections among partners and promotes men’s health. In Uganda, and many other settings, it is important for men, including men with HIV (MWH), to have children to meet personal, familial, and sociocultural expectations [1, 2]. Our group and others developed safer conception care programs to support HIV-affected couples to achieve reproductive goals and avoid HIV transmission [3–7]. In these clinical programs, many individuals and couples access services seeking support for unmet reproductive goals, often in the context of what is likely to be sub- or infertility.

Infertility affects 1 in 6 couples worldwide [8]. Primary infertility, when a person has never achieved pregnancy, affects an estimated 3% of Ugandans, and secondary infertility, when at least one prior pregnancy has been achieved, affects 35% of Ugandans [8]. Subfertility, or “any form of reduced fertility with prolonged time of unwanted non-conception [9],” affects approximately two-thirds of HIV-affected couples and may be associated with prolonged HIV exposure and transmission [10]. In societies that place high social value on having multiple children, all forms of infertility can be stigmatizing. Men and women with infertility often face stigma and discrimination in their partnerships, families, and social groups; intimate partner violence [11, 12]; and exposure to STIs and HIV [13, 14]. Yet, fertility services are limited to specialized centers in cities, while those in rural areas or without financial resources lack access to information and clinical services for couples facing subfertility.

HIV is linked to infertility bidirectionally [10]. Challenges meeting reproductive goals may influence sexual behavior, leading to more condomless sex and more sexual partners [10, 15]. Biological changes related to HIV, stress, concomitant illnesses, weight loss, and medications may also impact fertility [16]. Regardless of the etiology, people with HIV (PWH) who experience infertility may have limited access to reproductive health care as a result of high costs, stigmatization, and lack of specialized knowledge among providers [17].

Couples who have the option to engage in safer conception care may also face stigma for decisions to have children in an HIV-affected partnership [18, 19]. HIV stigma is intertwined with stigma related to having few or no children in this setting where HIV prevalence among men is 4.3%, the fertility rate is 4.6 births per woman [20], and an estimated 5–7% of couples married or cohabitating are serodiscordant [21].

Health-related stigma, characterized by social experiences such as exclusion, rejection, or devaluation resulting from judgment about a group with a particular health concern, impacts health behaviours and outcomes [22]. Examining stigma from an intersectional lens contextualizes the experiences of people with multiple stigmatized identities and improves understanding of how these interacting factors impact health-related decisions [22]. Understanding the intersectional stigmas experienced by Ugandan men and women affected by HIV who are trying to conceive is crucial to developing tailored educational content and interventions that promote conception, address subfertility, and ameliorate stigma experiences, while striving to prevent HIV transmission.

Limited research explores intersecting stigmas among HIV-affected couples experiencing sub- or infertility. Women’s experiences with childlessness have been explored, but gaps remain in understandings of couple’s and men’s experiences, especially in HIV-affected partnerships in which men and women may also face other stigmas related to reproductive and sexual health. Prior qualitative research in Uganda revealed that HIV-affected couples desire to meet reproductive goals while maintaining partnerships and preventing HIV transmission [2]. Other studies in sub-Saharan Africa describe individuals finding partners beyond their primary partnerships to achieve important sociocultural reproductive expectations [23, 24], potentially engaging in sexual behavior that increases HIV transmission and acquisition [25, 26]. In Rwanda, women who were in a secondarily infertile relationship experienced increased incident HIV infection, and men in couples experiencing infertility were more likely to report additional partners than men meeting their reproductive goals [27]. Lack of research on the individual, couple, and sociocultural drivers and impacts of these intersectional stigmas leads to a gap in safer conception counseling and care, failing to meet the multi-faceted needs of HIV- and infertility-affected couples.

We conducted a mixed-methods research study in rural Uganda to explore intersectional HIV- and subfertility-related stigma experiences of MWH and their female partners.

## Methods

### Study Setting and Design

This sequential mixed-methods Qual-Quant study was conducted in Mbarara, Uganda, a southwestern city with population of around 65,000, located approximately 275 km southwest of Kampala. Mbarara had an estimated HIV prevalence of 13.1% (among people ages 15–49) in 2020 [28]. The study explored intersecting stigmas related to infertility and HIV, pathways of sexual risk for HIV transmission, and other barriers to and promoters of HIV care and prevention among MWH and a subset of their partners. The quantitative component recruited 100 MWH with (*N* = 50) and without (*N* = 50) subfertility experiences. This manuscript provides an in-depth report on the qualitative component, in which we recruited 30 men with unmet reproductive goals and a subset of their female partners. We conducted individual in-depth interviews exploring intersecting infertility and HIV-related stigma experiences, factors influencing sexual transmission of HIV, and other barriers to HIV care and prevention among Uganda men and their partners.

### Study Population and Sampling Strategy

The study population included Ugandan men aged 18 years and older with HIV who reported trying to conceive with a female partner for at least one year with no known partner pregnancy. Men were approached by trained research assistants for potential study enrollment while attending routine HIV care at a regional referral hospital. Those who were interested in participation answered a series of questions to establish eligibility; eligible men participated in an informed consent process and the interview was scheduled for those who consented.

A subset of male participants’ female partners were separately enrolled. Women were invited via invitation letters delivered by enrolled men. Inclusion criteria included: being at least 18 years of age, partnered with an enrolled man, and knowing their partner’s HIV serostatus (to minimize inadvertent disclosure through study participation).

### Data Collection Methods

**Fig. 1:**
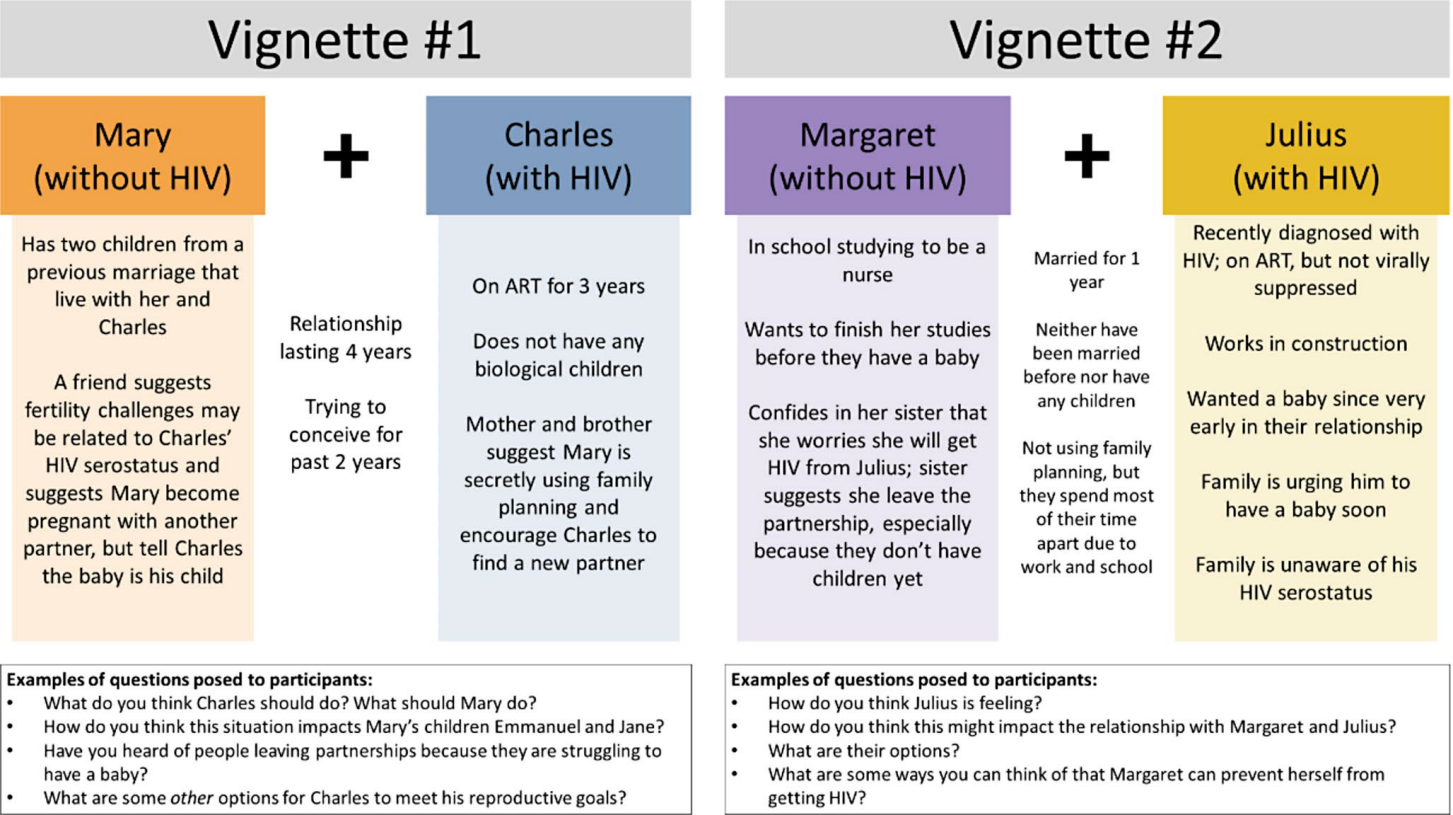
Fictional vignettes were used to frame questions about couples and scenarios dealing with issues related to HIV and sub-fertility

In-depth interview guides were developed to explore experiences of HIV and infertility stigma, informed by prior data [2–4, 29], the literature [30–32], and input from our research team, including local research assistants, research coordinator, and site principal investigator. Interview items explored partnerships and their characteristics; HIV serostatus disclosure; plans for and motivations to have children; medical history; access to clinical or other fertility support; knowledge and use of safer conception strategies; and family, community, partnership, and internalized stigma related to HIV and infertility. Fictional vignettes about Ugandan, HIV-affected couples were written based on prior research that described intersectional HIV and fertility stigmas [2] and served as foundations for discussion for participants who might have been hesitant to discuss their personal experiences (Fig. 1). All participants were asked all interview guide main questions, including those related to the vignettes, and interviewers probed based on outlined probing questions in the interview guide. Guides were translated to the local language (Runyankole) and backtranslated with modifications as needed by four Ugandan research team members fluent in English and the local language. Interviews with men and women were conducted separately by research assistants with undergraduate-level education and qualitative methods training; research assistants (EK, BRK) were supervised by the local research coordinator (MMO) who holds a Master of Public Health degree and has experience leading this team in recruitment and retention of men [2, 4, 7, 13, 14, 29, 33–35]. Each interview session was conducted in a private room, with only the research participant and the interviewer present. Interviews lasted between 40 and 150 min (mean 71 min, median 65 min).

Trained research assistants conducted the in-depth interviews in a private research space. Interviews were conducted in English or the local language (as preferred by the participant), audio-recorded, and later transcribed by the RAs. Final transcripts were reviewed for quality, consistency, and accuracy. Participants were assured their discussions would not be shared with their partner. Participants also completed a brief demographic questionnaire.

### Analysis Methods

We used content analysis to analyze interview data [36, 37]. Each transcript was read by at least three team members (MCP, MMO, JMT, ATM, ECA, LTM) and emergent constructs were categorized into coding nodes. The codebook was assembled, discussed among the analysis team and RAs who conducted the interviews, and revised several times. The final codebook was entered into NVivo analysis software where a subset of interviews were coded by multiple team members to ensure understanding and consistency of coding between team members. The remaining transcripts were coded by four individual team members.

### Human Subjects/Ethical Review Information

All participants were informed about the study and provided their signed consent prior to participation. The study protocol was approved by the Institutional Review Board at the University of Alabama at Birmingham, Alabama, USA (IRB-300008016), the Mbarara University of Science and Technology, Mbarara, Uganda (MUST-2021-213), and the Uganda National Council for Science and Technology, Kampala, Uganda (HS1964ES).

## Results

**Fig. 2:**
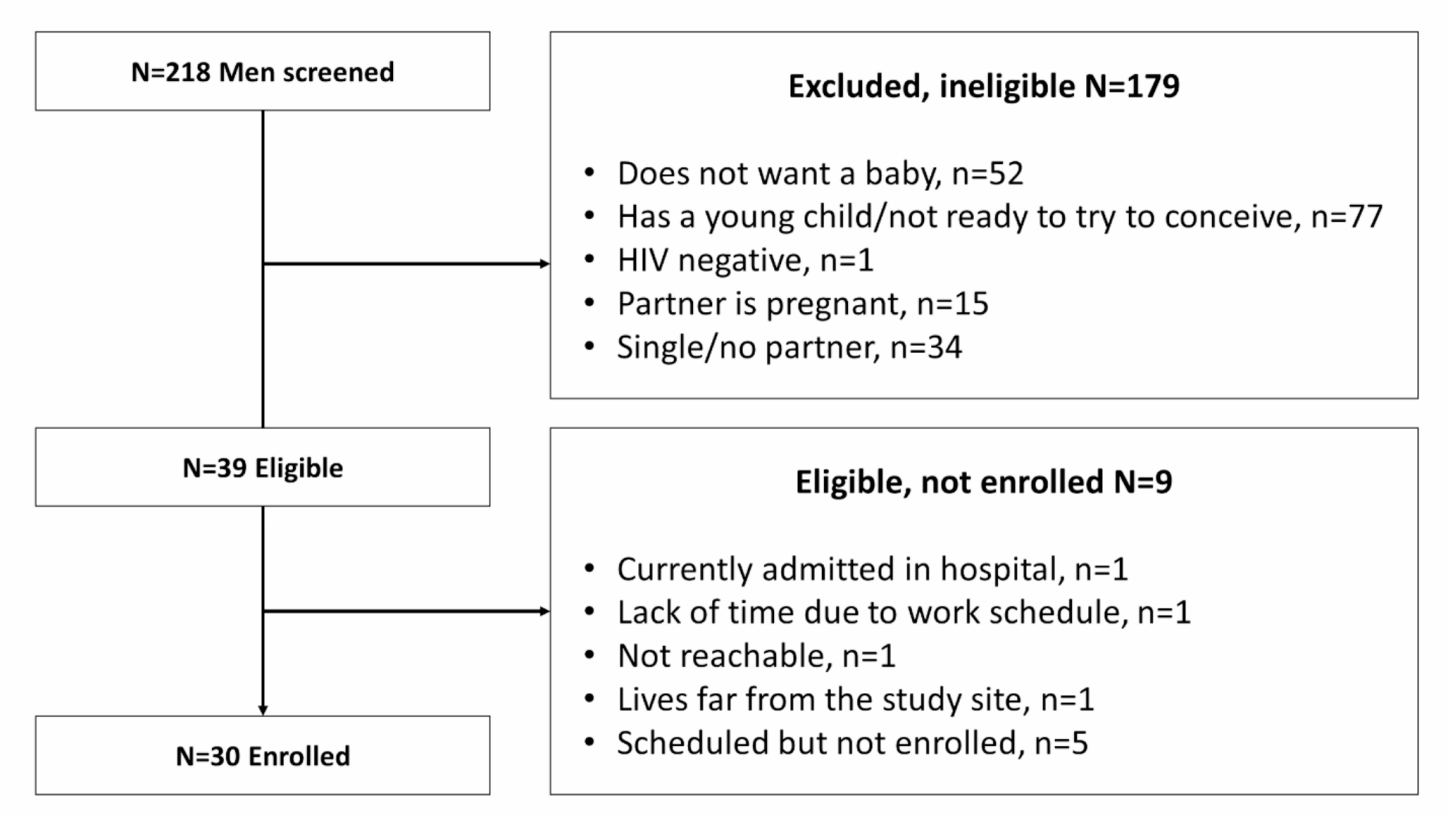
Number of men who were screened, eligible, and enrolled in the qualitative study aim

Two-hundred eighteen men were screened, 39 men were eligible (Fig. 2). Thirty enrolled men had a median age of 39 (range 25–75) years. Most were self-employed (*N* = 20, 67%), nearly all had at least one child under their care (*N* = 29, 97%), and had fathered at least one child (*N* = 28, 93%) (Table 1).

Ten participants’ female partners (median age 35, range 24–49) participated in a separate in-depth interview. Most were self-employed (*N* = 8, 80%), nearly all had at least one child under their care (*N* = 9, 90%), and had given birth to at least one child (*N* = 8, 80%).

**Table 1:** Demographics of male index participants and female partners

Variable	Male Index Participants N = 30 N (%)	Female Partners N = 10 N (%)
**Age**		(*Missing = 1*)
Median, range	39, 25–75	35, 24–49
**Country of birth**		
Uganda	30 (100%)	9 (90%)
Tanzania	0 (0%)	1 (10%)
**Highest level of education**		
Primary or less	18 (60%)	6 (60%)
Some secondary	8 (27%)	4 (40%)
Completed secondary	1 (3%)	0 (0%)
Tertiary/Vocational	3 (10%)	0 (0%)
**Employment status**		
Self-employed	20 (67%)	8 (80%)
Full-time employed	8 (27%)	1 (10%)
Part-time employed	2 (7%)	0 (0%)
Not employed	0 (0%)	1 (10%)
**Estimated monthly household income (past 3 months)**		*(Missing = 3)*
Median	$63.70 USD	$13.85 USD
Range	$0 - $692.37 USD	$2.77 - $108.01 USD
**Number of Children** (Median, range)		
Number participant financially supports (under age 18)	3.5, 0–8	3.5, 0–6
Number fathered (alive & deceased)	3, 0–11	N/A
Number of live births	N/A	2, 0–4

### Qualitative Findings

Men and women in HIV-affected partnerships were eager to discuss their reproductive goals in the context of fertility and HIV prevention. Participants were open about their experiences with delayed conception, pressures to have children, sexual behaviors, and HIV disclosure and prevention. They described experiences with multiple types and levels of stigma that make trying to conceive in an HIV-affected partnership challenging. We identified three overarching themes: (1) Reproductive goals often take priority over HIV prevention among HIV-affected couples in this context, influenced by internalized, anticipated, and enacted subfertility stigma in society. (2) MWH may pursue behaviors that increase HIV transmission risks in order to meet reproductive goals. (3) Men and women are eager to maintain their primary partnerships, prevent HIV transmission, and meet reproductive goals with guidance from healthcare providers. Despite these experiences, the interviewed men and women described a sense of resilience and commitment to their partnerships, at times highlighting love and loyalty as a powerful motivation for maintaining partnerships. In other partnerships, varying experiences of stigma influenced communication and intimacy breakdown. All participants desired accessible healthcare to help them meet their reproductive goals.

### Theme 1 – Reproductive Goals Often Take Priority Over HIV Prevention among HIV-Affected Couples in this Context, Influenced by Internalized, Anticipated, and Enacted Subfertility Stigma in Society

#### Motivations for HIV-Affected Couples to Have Children

Participating men and women were eager to have a child as soon as possible. They described complex decisions encompassing multiple aspects of their sexual and reproductive health decision making, including finances, loyalty, HIV prevention, future planning, fertility, fidelity, and other reproductive health concerns. Participants expressed desire for counseling that addresses multifaceted needs while planning for pregnancy.

[My husband] always talks about how I don’t want to have children for him, and that I seem to have other men, and all that is because I don’t want to have children yet … He told me that he is getting weak due to HIV and that he’s getting older, so he needs to have more children … He’s 37 years old. He said since he’s on drugs, his life keeps on decreasing like he knows he will die. But we work for people … he doesn’t have a plot of land that I can raise the kids from once he is gone, so you wonder what to do when you have more children and he’s no more.” – 012 Female Partner.

Why would you need an income when you do not have children or why would you go ahead to work? … When you have a child, you must plan for her future and how she will survive when you are no longer in this world. You have to leave a foundation from where he or she will start. – 013 Male Participant.

#### Pressures on Men to Have More Children

Having children informs social standing in Uganda. For men, children can indicate that one has worked hard and can care for a family. Male children are valued as inheritors of the family lineage and property, while female children are valued as caretakers in the home and a source of wealth in terms of bride price. In our sample, men who had children desired more children.

Now if you have not given birth then where do you put yourself? I have an uncle that married 4 women but they never gave birth. He sent one away and got another like that but failed; so we don’t count him as a man. If you want to see a family then it should have man, wife and children so without that we don’t count you, in fact you’re just ‘half’ [says it in English] and we see you as a person in trousers; you’re in an image of a man but not a man … When growing up, they would show us an abandoned house and tell us, ‘You see that house, it was for a man that didn’t give birth and died.’ … A person that has never given birth but died is not easy. His ghost can come and disturb you, especially other children… – 008 Male Participant.

MWH reflected on their perception that they could die soon and the importance of having children before death. This internalized pressure on men to meet their reproductive goals quickly also put pressure on their partners to help them meet these goals.

When you have your things, … you must plan for them. What if I die, who will take care of them? This is the reason why you need to have children that you have well trained in case you die they take care of those things or even add more.” – 04 Male Participant.

One day I asked [my husband] about what causes him to be sad and … he said that ‘Us people with HIV, we can die anytime and I am going to die without having a child, who will I leave behind?’ He also said that not having a child has left him with a wound in his heart. That’s the reason why you can never find him happy. – 007 Female Partner.

Men may also face partner pressures to have [more] children, as women worry for their own social and economic security in partnerships that are not stabilized with shared children.

Well, one child would be enough but I really love my wife and when she doesn’t have a child with me, it makes her think a lot. She thinks that the house is not hers, she would be sent away if I died … it’s easy for one to leave the family if she hasn’t had a child… It’s because I have an older child who’s a boy. That’s why I want her to have children so that she can feel at home, raise her children and have a belonging. – 027 Male Participant.

#### Pressures on Female Partners to Produce Children Even in the Context of HIV Exposure

For women, maintaining a partnership, bearing children, and meeting male partner needs are socio-cultural norms. While women are concerned about HIV acquisition, their sexual health decision-making power is limited by their need to maintain partnership-related social and economic security. They described expectations to maintain their partnerships, but partnerships affected by HIV evoke community stigma, leaving women in this position feeling powerless.

If a woman doesn’t give birth to children, she cannot stay in that home. In our village, there are some men who … chase their women out of houses … If a woman doesn’t have children, she will get fed up and leave and if a man doesn’t have children with a woman, if he gets fed up with her, she will leave. Children are the ones that hold us in our families. – 029 Female Partner.

Women, all of whom were partnered with MWH, described additional community stigma and pressures related to preventing transmission of HIV to their children.

The [community] always gossips … ‘Why does this sick person want to have children yet he’s already sick?’ … Most of them don’t have an idea that a sick person can give birth to an HIV-negative child. – 009 Female Partner.

Women feel like there are few safe spaces to ask for help, even from other women, as bringing up these topics may unintentionally disclose the experiences of HIV and subfertility.

There is a woman in our village I would want to ask how she was able to give birth… That woman is my neighbor and … she is still giving birth with a man who they say has HIV… Here at the hospital, it’s not hard for me [to seek guidance on having a child with a partner with HIV] because right now no one knows that I am here… but waking up one day and I ask my neighbor to explain whether the children she is giving birth to have HIV or asking her what they did for her to make sure that she gives birth to children who do not have HIV—it might be very difficult for me. – 004 Female Partner.

#### Negative Effects of Inter-Related Subfertility and HIV Stigma

For both men and women, unfulfilled societal expectations around fertility contribute to negative self-image, fear of rumors and peer opinions, and experiences of discrimination and abuse.

When you are young and get married and fail to get children, the family members of the man will say that the woman he married is barren because no one has ever accepted that their son can be barren. It’s always you [woman] who has come from another family that doesn’t give birth. Or they will say that the woman is aborting the pregnancies and is not able to produce for them a child. – 004 Female Partner.

Women without children are accused of clandestine use of family planning or mistreatment of others’ children. They are compared to transactional sex workers, another stigmatized identity.

[People in my community] say, ‘A woman came to sleep with the man, she came to just eat,’ and those words are very painful, so you wish to produce one child at least. Sometimes you hear people say, ‘Oh, you mean the man with a woman that doesn’t give birth,’ so you hear that, as much as you make yourself feel strong, but you feel pain. Most people think that someone who didn’t give birth has no mercy on other children… – 005 Male Participant.

Don’t you know about women? She will ‘compete’ (says it in English) with the first wife who has children. She will think she has to give birth to children with me… She knows that whoever has children is a woman. Every woman knows that whoever has given birth in the family is the one called a woman so if you don’t have a child then you’re like a prostitute and that disturbs her. – 008 Male Participant.

Participants explain how experiences of HIV stigma in partnerships and society can be discouraging for maintaining partnerships with few or no children. Cultural pressures to have many children conflict with local taboos related to being in an HIV-serodifferent relationship.

I was really scared about how to live with [my husband] in that situation of HIV … When I returned to his home, I also pictured leaving my children alone, I was stuck. I looked at the house we had built together because I found him when he didn’t have a house; we worked together and built one. I looked at the land we had bought and the things we had accumulated together and I got confused. I just decided to stay there with him but in staying together with him, every time we are having sexual intercourse I am scared, and I always think that he will infect me but I do not have a solution… I have two girls with this man and now he has started asking me why I do not give him a boy … This is a problem I have now and when I look at my beautiful girls… I love them so much so whenever I think about leaving them, I get hurt. Even if I take them, I cannot manage looking after them. I cannot leave them neither can I manage them alone, so I have decided to be patient and if this man infects me, I will die and my children will bury me… I think about the failure to have another child and I think about getting HIV… – 029 Female Partner.

Some partnerships are maintained, but experiences of HIV stigma influence broken-down communication and intimacy.

My wife thinks that if someone has HIV, [they] might not be able to have children. That is how she thinks. Before we got HIV, she was trying to see that we meet often trying to get pregnant, but now the situation is bad. I have to first beg her to have sex with her. It always feels like I am starting on a fresh page with a new person. Sometimes I can even go for like three months without having sex with her, yet we sleep in the same bed. – 018 Male Participant.

### Theme 2 – Men with HIV in Uganda May Engage in Behaviors that Contribute to HIV Transmission in Order to Meet Their Reproductive Goals

#### Additional Partnerships

Men described pursuing partnerships and marriages outside of their primary partnership to meet their reproductive goals, including having their desired number and gender composition of children, or “testing” their fertility.

I had mentioned that my first partner and I had tried for 6 years [to have a child] but failed, so I wondered if it was I that couldn’t have children. I decided to go out of the marriage and have a relationship; in so doing I had a child with that second partner. I realized I can have children so we had 3 children together.” – 030 Male Participant.

After trying with my wife to get a child and failing, I decided to get another woman and marry her. I rented for her a house in town, stocked for her everything… I explained to her the reason I had married her and she accepted. I told her that the reason I had got her was not to use her but I wanted a child. Because I told her that if she wasn’t ready to give me the child, we should cancel the relationship. – 007 Male Participant.

Women also alluded to their partners looking to conceive outside of their partnership, while feeling like they could not do the same.

I cannot go out of the marriage to test myself and see if he can have children or not. – 018 Female Partner.

Participants expressed suspicions about acquiring HIV from alternative partners while trying to meet their reproductive goals or test their fertility.

I am very sure that he acquired the sickness while trying to look for a child… He went out of the marriage to have a child so that’s how he got HIV. – 018 Female Partner.

Reactions to the vignette describing Mary and Charles’ relationship (Fig. 1) and advice to seek additional partners for both male and female characters show that societal norms allowing men to have multiple partners do not extend to women. Some men were supportive of Charles seeking another partner to conceive with, while women and other men disagreed with the advice.

Don’t you see it now? It means that when you do not have a child, they will chase you away. But now, me, I am not using family planning. That I am sure about it. Why don’t I get help and be checked and told why I do not get pregnant… Charles should first seek for treatment for his wife. He can do some investigations on whether she is on family planning. But can a woman get married to the man she loves and then she fails to give him children? No. Charles should first find out why his woman is failing to have children and after knowing this, he can then make a plan. He cannot just chase her away. What if it’s him who cannot have children? It’s like how my man can marry another woman because I have produced only girls. The woman he marries can also produce only girls like I did. He should first find out the reason why we have produced only girls or why we do not have children at all. – 029 Female Partner.

Participants talked about distrust between partners, clandestine use of family planning, and the negative consequences of women seeking additional partners.

I think Charles is like me. Many people have told me the same saying that my wife could be using family planning and she is just fooling me, she doesn’t want to give me children. They also told me that maybe she could have had children back at their home and she is hiding from me… And about what they told him to marry another woman, I can also do the same but how do I do it? I want it because I want a child. Let me first finish paying my loan then I will see about getting another partner… If [Charles] has the capacity, he can go out, elsewhere and get a child. That’s how I see it. – 007 Male Participant.

Participants also discussed the complexities of seeking additional partners when living with HIV and the maintenance of partnerships with mutual HIV disclosure. They offered suggestions that Mary and Charles should talk about their delayed conception together to find a solution.

What I think about this conversation, it has also happened to me and I first thought that my partner could be using some tablets [oral contraceptives] but hiding from me. But I slowly investigated and I found her innocent. It happens, women—you are dangerous… you hide secrets. A woman can dodge you when she doesn’t want you to have a child with her. Because you see about Mary and Charles, Mary came with two children; maybe she hadn’t also understood why Charles should have a child with her. You might find her two children are making her suffer, so she is wondering how she will take care of more children from Charles. This will add on her burden like these pictures I always see men carrying the whole family. So it depends. A woman can use family planning when she doesn’t want to produce with a man. It happens. Like we had talked above when a man suffers from HIV and hides it from the woman. It is not good. And these people are there. A man gets infected with HIV, but they get to know at a point of dying when he hadn’t told the wife. It is not good… If Mary is using family planning, she is betraying her husband because she only wants Charles to feed her, but she doesn’t want to produce for him. And for us men, we love women as our gifts. We love you; but we want you to give us children. – 004 Male Participant.

#### Process and Timing of Disclosure

Serostatus disclosure to primary partners was an inclusion criterion for this study; yet many men and their partners described incomplete and delayed disclosure. Men described experiences of rejection after HIV serostatus disclosure and therefore felt discouraged from disclosing to future reproductive partners.

If I was to marry, I would have married [a long time ago], but … whenever I’d talk to a woman about marrying her, she’d say we should go and test [for HIV], so I would fear, going to test yet I know that I am sick, will I continue going testing? No, so once I have left them, I wouldn’t go back to that person. Going to the next person, she would tell me the same, so I just left all that and left marrying things. With this partner I have, I got her as a blessing, it’s like God sent her for me. So many women didn’t want to marry me because I have HIV. – 003 Male Participant.

Men in our sample delayed or indefinitely postponed serostatus disclosure to their partners out of fear of abandonment, especially when they did not have children with their partner to secure the relationship.

We met in our field of work and started a relationship. Later I married her and we started staying together. She didn’t know about my HIV status then. After living with her for some time… [silence]… I am not the one who told her about my HIV status in the first place. It was very hard for me to tell her. I was always thinking about telling her but I couldn’t … Looking at a girl you love, who you have started a life with and knowing that if you tell her that you have HIV, she will leave you, I was really scared. If someone has not had children with you, she can leave… I have seen people’s marriages failing because of that. Someone marries a wife, when she gets to know that the husband is sick with HIV; she goes to test and if she finds herself HIV-negative and having no children with that man, she decides to leave him. – 009 Male Participant.

Participants also described pressures from family to prioritize reproductive goals over HIV serostatus disclosure.

Actually, some of [my family members] tell me that I do not need to disclose to the woman about my status; they tell me to just go and impregnate any one and I get a child. I feel like I have disappointed them because I think they might think that I am barren. – 002 Male Participant.

Partnership decisions related to HIV disclosure, safer conception practices, and reproductive planning appear to be driven primarily by male partners. Men decide if and when the couple will use condoms, discuss HIV prevention, and get tested for HIV.

He didn’t tell me at first [about his HIV status]. but I came to know about it when I saw the drugs… He’d ask me why I am asking about private things. When I became pregnant, he sat me down and told me to test for HIV … because he had HIV … I was negative while he was positive. I was really scared. We were counseled, we started using condoms, but I was already pregnant. I really hated myself and thought I was going to die. It was so hard for me to tell my family, so I just did when I was going to have my second child which is 10 years after that. That really hurts me even if we use a condom, since he’s an alcoholic person, he will not accept to wear a condom all the time so that ‘tortures’ [says it in English] me although I am now used to it and much better.” – 009 Female Partner.

He came here at the hospital and when he went back home, he told me that we can start using condoms… We had never used a condom… He did not tell me the reason why they taught them like that. He only told me that we were going to start using the condom and he stopped there. – 001 Female Partner.

Women discussed the disproportionate lack of information and education about HIV that they receive compared to their male partners with HIV. MWH receive counseling and education on HIV transmission and prevention when presenting for treatment at the local hospital, while women without HIV partnered with a MWH may only have the knowledge that their partner chooses to share. This imbalance of information was also clear in quotes from men.

The main thing [my partner] tells me is, ‘If you disorganise yourself, you will get HIV.’ With that he means that if I sleep with other men, then I will have HIV. His doctor once told me that if the man is taking his drugs well, he might not infect the partner but I don’t know if that is very true.” – 012 Female Partner.

[Our relationship] has not been good because of failure to have children … We lived together for four years before I knew that he had HIV. He would … get his medicine refilled but he would keep it at his brother’s place. Then he would keep a few tablets in his jacket which he would swallow in the morning before he leaves for work … One day, he left that jacket at home. I didn’t know how the HIV tablets looked like but when I saw them, I got frightened … I asked him that if he knows that he is sick he should also take me to hospital for testing such that I can also start the treatment in case I am found infected. He denied and said that those tablets were medicine for chickens … I begged him to tell me … That’s when he narrated the whole story to me. After telling me, I was hit by pressure and I fell sick. I hated myself… I thought about how I was going to go to the hospital, get tested and be told that I had HIV. I knew I would die there so I didn’t go to test. I ignored it. It was after a period of about three months, I got a small papule on my breast and I told him that these could be the signs of HIV and begged him to take me to the hospital for testing. He didn’t. Another one (papule) hit me on the back… That time around I told him that I was not going to die without knowing my status… He then brought me to the hospital, I tested and I was told that I had HIV. – 007 Female Partner.

### Theme 3 – Men and Women are Eager to Maintain Their Primary Partnerships, Prevent HIV Transmission, and Meet Their Reproductive Goals with Guidance from Healthcare Providers and Community Supports, like Couples Counseling

#### Seeking Clinical and Non-Clinical Care

Men and women expressed that they wanted to learn more about their individual and partnership reproductive capabilities and discussed how they had sought assistance. Participants described the costs they have endured seeking fertility care.

I have not had the capacity all this time. I haven’t got money to look for a child or go to the hospital to check my eggs. I surely do not have this money. After coming to participate in this study, I am hopeful that in case it continues on to the extent of bringing us machines that would check us, I would get tested and see if I can have children … I have drunk herbal medicine and I have visited the witch doctors, but I have failed. In case I get money, I will be able to visit a doctor and check if I have the eggs. And if I have them, then he will give me a way of how I can get pregnant and if he says that I do not have the eggs, it’s still fine. I will not kill myself… – 007 Female Partner.

I was planning to come to the hospital to see the doctors because I saw people with HIV that gave birth to very beautiful children, that’s when I came to [counselor] and he told me, ‘No, you will give birth, bring your wife’ … She was told what to do and was given drugs; so that was about 6 years ago. During that time, she had just gotten her operation where they had removed the blood that was in her [fallopian] tubes, so she failed to become pregnant when she was given the drugs … She came to [counselor] and her uterus was tested and found with an infection. She was told to bring 85,000 UGX [~ 22 USD], which I brought, after that she was told to bring 125,000 UGX [~ 33 USD] for the water that was in the tubes to be removed… I told them I needed time to get that money; so up to now, it’s what I’ve been looking for… The support we want is what we are looking for in doctors. We haven’t got any other support. – 006 Male Participant.

#### Love Can Build Resilience

Participants described how love, commitment, faith, and shared experiences influenced their decision to stay in a partnership, despite challenges associated with HIV and fertility.

I tell you that love is an important thing. To get a partner and you tell her that you have HIV and she accepts to sleep with you, you see that her love is beyond. And you tell her that let’s have a child through a good way and she accepts to take part, you see that she has really accepted to do this thing. – 004 Male Participant.

I hear people say that my man was tested and found with HIV and I remained with him, and I wonder what people think. I think it’s love. Love is greater than everything.” – 004 Female Partner.

Yes, because we have gone through so much and I love her… We tried for 6 years and finally had this one child, then tried after having the child, so we have been together for 10 years… At some point, she hated herself and wanted to leave the marriage, but I counseled her; told her that I am the one that would chase her since she hasn’t had another child, but once she knew my thoughts to that, she was okay… It wasn’t easy for me because I had the desire to have children … I was not going to be fine if she left because I suffered with her which caused me to love her more as she was very patient with me and my sickness. So, I decided to stay with her… Whether I have children or not, that’s God’s will but as long as we stay together. – 030 Male Participant.

#### Calling for Comprehensive and Affordable Reproductive Care

Couples affected by HIV want to discuss their reproductive goals with healthcare teams to receive knowledge and clinical support, but the lack of access to services is a barrier, along with barriers of internalized and community stigma related to both HIV and subfertility.

I think that he will marry or else get this child outside our marriage because he really wants the child … I wish I can get money to go to the hospital and get checked and treated… I want a person who will tell me how I will live with my husband without getting HIV. That is if I am not yet infected [laughs]. That person should first test me and see if I am still okay, then he tells me how I should do it to make sure that I do not get HIV. Or he tells me that he can give me some medicine to help me get pregnant or he checks me and finds out why I am not getting pregnant and he treats me… It can be easy if someone offers to check me and treat me now without asking for money because I do not have it… The problem is money. That is what is killing us, poverty. There is no money to pay a doctor to help treat you and you get a child. We are forced to stay in our villages digging [subsistence farming] without any solution. No money, no going to test for HIV. Our nearby health centers do not test for HIV and you fear going to the clinics because the people you find there will talk about it because they know you. So, you stay in the village with your problems as you watch years pass by. – 029 Female Partner.

Participants hoped to receive additional counseling from the study team, including referrals to affordable integrated HIV and fertility care and guidance on next steps to meet their goals.

If you have the same health service provider, giving you HIV treatment, helping you and your partner to have children and makes sure that your partner doesn’t get infected with HIV, I see that might be more beautiful than moving from one person to another. If it’s the same person, he will make you understand more and better. – 009 Male Participant.

[Talking to a healthcare provider about my reproductive goals and HIV prevention] is what I would love. If someone did that for me, I will be reborn. He will have taken these two things away from me; shame and being disrespected. It would make me feel born again. I might love that person forever until I leave this world … I will feel born again; I will feel like a baby. – 007 Male Participant.

## Discussion

Qualitative interviews with Ugandan MWH and a subset of their female partners revealed experiences of intersectional stigmas related to unmet reproductive goals in HIV-affected partnerships. Our data suggest that societal HIV and infertility stigmas are widespread in this community, and our participants were eager to discuss their experiences and desire to receive services to address their HIV prevention and fertility needs.

Participants described complex intersections of HIV and infertility stigma experienced differently by men and women. Women partnered with a MWH may prioritize having children over HIV prevention because their social standing and financial well-being are deeply connected to maintaining partnerships. Limited decision-making power and HIV prevention knowledge is shared with women, causing them to feel stuck between two stigmatized situations: a partnership affected by HIV and a partnership with no children. MWH face HIV-related stigma that affects their ability to find a partner to conceive with. As they seek additional partners to meet their reproductive goals, disclosure of HIV serostatus to primary and additional partners is often delayed, incomplete, or entirely absent.

While this sample of MWH had not been discouraged from accessing HIV care, the disproportionate balance of power and knowledge between male and female partners indicate a greater need for couples-based counseling and integration of HIV prevention strategies for women (e.g., PrEP). Despite facing intersecting stigmas related to HIV-affected partnerships and unmet reproductive goals, men and women were eager to maintain their partnerships, and draw on shared experiences, love, and external supports to persevere in their relationships and reproductive journeys. Men and women want to speak with healthcare providers about their sexual and reproductive health needs, including STI and HIV testing and treatment, HIV prevention, subfertility, and trying to conceive.

Research examining the intersections of HIV- and fertility-related stigmas is scarce. Analyses of HIV stigma intersecting with other sexual-and-reproductive-health- and gender-related stigmas provide helpful comparisons. Embleton et al. explored five case studies of adolescents in Uganda, Kenya, and Ghana who experienced intersectional stigmas related to their HIV status, gender, poverty, age, sexuality, and gender norms that affected HIV prevention and treatment uptake [38]. Drivers of intersectional stigma included fear of HIV infection, social and economic ramifications, judgment, blame, prejudice, and stereotypes [38]. Inequitable gender norms and relationship power dynamics drove stigma within these cases; yet social support mitigated their stigma experiences [38]. A metasynthesis of stigmas intersecting with HIV stigma in sub-Saharan Africa found that HIV- and gender-associated stigmas were the most salient [39]. Among men, this intersection of stigmas prevented HIV testing, care engagement, and disclosure to preserve traditional masculine ideals; among women, the risk of abandonment and loss of financial security were associated with these stigmas [39]. Our findings also show that threats to social and economic security prevent women from leaving partnerships, despite feeling vulnerable to HIV infection. The inequitable distribution of sexual, reproductive, and economic decision-making power in favor of men leaves women susceptible to social isolation, judgment, blame, prejudice, and poverty if their partnership is not secured through having children. Women’s power to negotiate safer conception practices is limited, especially in the face of pressures from family and community to have children soon after a partnership begins. Social support in the form of communication between partners, couples counseling, and integrated HIV and sexual/reproductive health care described by our participants may mitigate stigma experiences [40].

Additional emergent findings included experiences of partnership and community stigma influencing the decision to disclose HIV serostatus in partnerships, especially those that were new. Men discussed their trepidations about telling new partners or wives about their HIV status, as this was perceived as a barrier to meeting their reproductive goals. Additionally, both men and women described experiences with multiple sexually transmitted infections that they experienced and were treated for while trying to conceive, including gonorrhea and syphilis.

This study has strengths, including the strong rapport the local research team has with the community, which supported our participants to feel comfortable discussing culturally sensitive topics. Using vignettes to introduce these topics gave participants the opportunity to relate their own experiences or comment on shared experiences related to infertility and HIV. These long, detailed interviews explored infertility stigma with men—who are often excluded from these discussions in clinical settings—and engaged female partners to explore viewpoints from both genders.

Limitations include that men were recruited from the local rural hospital where they were accessing HIV care. MWH recruited from community settings who are not yet engaged in care may have different attitudes and experiences related to fertility and HIV. Female participants were limited to those invited by their male partners and knowledge of partner serostatus was an inclusion criterion. This study did not explore the clinical diagnostic factors leading to these infertility experiences, and participants were not clinically diagnosed as infertile. Thus, the experiences of unmet reproductive goals in this sample may or may not be related to clinical subfertility. However, as we learned, the perception of subfertility can be enough to influence sexual decision-making and HIV risk.

## Conclusion

In this analysis of in-depth interviews with men with HIV and their female partners trying to conceive, we found that men and women want to discuss their reproductive and sexual health care needs. They want to receive services for HIV care, prevention and fertility from the same location/providers. The importance of having children to maintain and strengthen partnerships may overshadow concerns about HIV transmission, especially among men on HIV treatment who have received counseling on treatment as prevention. Women’s lack of decision-making power and HIV prevention knowledge leaves them feeling trapped and vulnerable, presenting an important opportunity for PrEP services. Further research into the etiology of sub- and infertility for HIV-affected couples in East Africa is needed to understand how to best support HIV-affected couples trying to conceive. Furthermore, additional research exploring the intersections of HIV and infertility stigma are needed in settings with high fertility and HIV transmission rates to design interventions that address social, emotional, and medical needs of couples and individuals.
